# ﻿Chironomidae (Diptera) from mountain lakes of the Eastern Carpathians, Romania: First records and insight into diversity

**DOI:** 10.3897/zookeys.1233.142856

**Published:** 2025-03-28

**Authors:** Peter Bitušík, Veronika Slobodníková, Milan Novikmec, Adam Dudáš, Ladislav Hamerlík

**Affiliations:** 1 Faculty of Natural Sciences, Matej Bel University, Tajovského 40, SK-974 01 Banská Bystrica, Slovakia Matej Bel University Banská Bystrica Slovakia; 2 Faculty of Ecology and Environmental Sciences, Technical University in Zvolen, T. G. Masaryka 24, SK-960 01 Zvolen, Slovakia Technical University in Zvolen Zvolen Slovakia; 3 Institute of Zoology, Slovak Academy of Sciences, Dúbravská cesta 9, SK-845 06 Bratislava, Slovakia Institute of Zoology, Slovak Academy of Sciences Bratislava Slovakia

**Keywords:** Biomonitoring, high-altitude lakes, males, Maramures Mountains, new record, non-biting midges, *Procladius* identification key, pupal exuviae, Rodna Mountains

## Abstract

Lakes at high altitudes are extremely sensitive to environmental stressors at both local and global scales, making them important sentinels of the changing world. Chironomidae, the most diverse group of benthic macroinvertebrates inhabiting mountain lakes, respond to various environmental impacts, making them important bioindicators of the lake’s ecological status. This study aimed to provide the first insight into chironomid diversity in high-altitude lakes from two mountain ranges of the Romanian Eastern Carpathians: the Maramures, and the Rodna Mountains. Floating chironomid material was collected by skimming the water surface with a hand net from 16 lakes at elevations ranging from 1378 to 1922 m a.s.l. A total of 50 species/ taxa were collected, including nine new records for Romania. Notes on newly recorded species’ distribution, ecology and taxonomy are provided. In addition, an identification key for *Procladiuschoreus* and *P.sagittalis* based on thoracic horn characteristics is given. With our addition, the total number of chironomid species known from Romania is now 526. The study provides a baseline for future research on chironomid diversity, ecology, and biogeography in high-altitude lakes of the Carpathian Mountains.

## ﻿Introduction

Chironomidae is the most diverse group of benthic macroinvertebrates inhabiting high-altitude lakes and ponds, where they can represent twice the diversity of all other macroinvertebrate groups and often predominate quantitatively as well (e.g., Lods-Crozet et al. 2012 and references therein). Due to these attributes, chironomids are considered a good surrogate for benthic macroinvertebrates in ecological studies and biomonitoring programs (Ruse 2010).

The sensitivity of chironomid species to various environmental impacts, such as climate change, long-range air pollution, and species introduction, makes this insect group important bioindicators in both contemporary and palaeoecological studies (Nicacio and Juen 2015 and references therein).

Understanding the regional chironomid fauna of mountain lakes is the first step towards using the species for lake status assessment and further monitoring. Additionally, faunistic data from contemporary limnological studies can aid in the interpretation of paleolimnological data in mountain regions (Battarbee and Bennion 2012).

Our ongoing limnological research on chironomid fauna in alpine lakes of the Eastern Carpathians has shifted from the Chornohora and Svydovets Mountains in Ukraine (Bitušík et al. 2020, 2024) to the Maramures and Rodna Mountains in northern Romania.

In this study, we provide the first insight into the diversity of the Chironomidae family in mountain lakes of these ranges, including species recorded in Romania for the first time. The results will serve as baseline information for further research on chironomid diversity, ecology, and biogeography in Carpathian lakes, as well as a basis for long-term monitoring of lake ecological status and as a prerequisite for developing appropriate management and protection strategies for these ecosystems.

## ﻿Material and methods

### ﻿Study area and sampling sites

**Figure 1.**
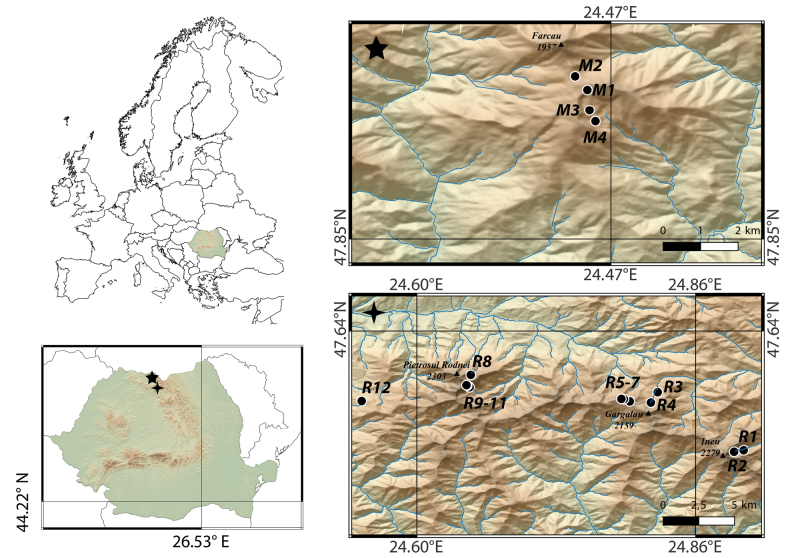
Geographical location of the study area and maps showing sampling sites in the East Carpathians. Star marks the Maramures Mts, spark denotes the Rodna Mts. Site codes correspond to the codes in Table 1.

The study was carried out on 16 lakes within two orographic units of the Eastern Carpathians in northern Romania: the Maramures, and the Rodna Mountains (Fig. 1).

Both massifs are extensive, covering an area of 1500 km^2^ with a length of around 100 km (Maramures Mts) and 1300 km^2^ with a length of almost 50 km (Rodna Mts). The Rodna Mts are the highest range in the Romanian Eastern Carpathians, with peaks exceeding 2200 m, while the Maramures Mts reach a maximum elevation of 1957 m.

The geology of both massifs primarily consists of crystalline rocks (gneiss, epimetamorphic schists, mica schists) penetrated by eruptive rocks (dacites, andesites, rhyolites) and sedimentary rocks (conglomerates, sandstone, clay schist, shale, marl, clay; Curtean-Banaduc et al. 2008; Chis 2010).

Both mountain ranges have a moderate temperate continental climate with Atlantic and Baltic influences. Based on data from the Iezer meteorological station (Rodna Mts, 1785 m a.s.l.), the mean annual temperature is 1.4 °C, mean air summer temperature is 9.4 °C, mean July air temperature is 10.3 °C, and the annual precipitation is 1240 mm, applicable to lakes at or above the natural timberline (1700–1850 m) (Kucsicsa 2011). Adjusted to 1360 m a.s.l., the forest zone has an average annual temperature of 3.4 °C, a mean summer temperature of 11.6 °C, and a mean July air temperature of 12.5 °C (Farcas et al. 2013; Diaconu et al. 2017).

The Rodna Mts were heavily glaciated during the Last Glacial Maximum and show clear glacial imprints, such as glacial cirques, lakes, and peatbogs (Mindrescu and Evans 2014). There are about 23 lakes in the Rodna Mts, each with a surface area under 0.5 ha and a maximum depth of 5.2 m (Chis 2010). In contrast, Pleistocene glaciation had a lower impact on the Maramures Mts (Costea 2008). The lakes of glacial origin there are generally small, and many are in an advanced terrestrialization phase or have already turned into peat bogs (Chis 2010).

The surveyed lakes in the Maramures Mts include four lakes situated in the Farcau area (Chis 2008) at altitudes ranging from 1603 m (the lower lake in the glacial cirque Vartopul Mare) to 1786 m a.s.l. (Lake Livia). The 12 studied lakes in the Rodna Mts are located at altitudes between 1378 m (Lake Taul Muced) and 1922 m a.s.l. (Lake Lala Mica) (Table 1).

**Table 1.** Basic characteristics of the sampling sites. If we were unaware of official lake names, we named the lakes for the adjacent hills. Maximum depth was either measured in the field or abstracted from available literature: *Akinyemi et al. (2013), **Mindrescu et al. (2013). Lake area was estimated using the polygon tool in Google Earth Pro. +/- indicate presence/absence of inflow/outflow. Abbreviations of catchment characteristics: **EAP** – extensive alpine pastures, **AM** – alpine meadows, **DP** – dwarf mountain pine shrubs (*Pinusmugo*), **RS** – rhododendron shrubs (*Rhododendronmyrtifolium*), **NS** – Norway spruce (*Piceaabies*), **BR** – bare rocks, **P** – peatbog.

Mountain range/ Lake name	Code	Geographical coordinates	Elevation (m)	Max. depth (m)	Lake area (m^2^)	Inflow/ Outflow	Catchment
**Maramures Mts.**							
Livia	M1	47°54.26'N, 24°27.59'E	1786	3.5	622	-/-	EAP
Vinderel	M2	47°54.55'N, 24°27.33'E	1677	5.0	6183	+/+	EAP
Vartopul 1	M3	47°53.82'N, 24°27.63'E	1647	0.8	843	+/-	EAP
Vartopul 2	M4	47°53.59'N, 24°27.76'E	1603	0.8	246	+/-	EAP
**Rodna Mts.**							
Lala Mare	R1	47°31.68'N, 24°53.99'E	1805	1.5	6665	+/+	AM, DP
Lala Mica	R2	47°31.60'N, 24°53.46'E	1922	0.6	5332	+/+	AM, RS
Taul Hardau	R3	47°34.93'N, 24°49.20'E	1552	*3.5	568	-/-	EAP, NS
Taul Stiol	R4	47°34.35'N, 24°48.82'E	1657	5.0	10790	+/+	EAP, DP
Gargalau 3	R5	47°34.42'N, 24°47.64'E	1911	0.9	263	-/-	EAP
Gargalau 2	R6	47°34.55'N, 24°47.32'E	1887	1.2	120	-/-	EAP
Gargalau 1	R7	47°34.56'N, 24°47.18'E	1894	0.4	601	-/-	EAP
Lacul Iezer	R8	47°35.90'N, 24°38.78'E	1822	**4.5	3883	+/+	AM, BR, DP
Buhaescu 1	R9	47°35.21'N, 24°38.71'E	1825	3.0	783	+/+	AM, BR, DP
Buhaescu 2	R10	47°35.30'N, 24°38.58'E	1892	5.2	1529	+/+	AM, BR, DP
Buhaescu 3	R11	47°35.33'N, 24°38.58'E	1911	2.0	659	-/+	AM, BR, DP
Taul Muced	R12	47°34.44'N, 24°32.70'E	1378	1.0	593	-/-	P, NS, DP

Except for two forest lakes (Taul Muced, Taul Hardau), the remaining lakes are above the recent tree-line, averaging about 1600 m in the Rodna Mts (Kucsicsa 2011). The natural tree-line has been significantly impacted by deforestation and grazing, lowering the upper forest limit and fragmenting the transition between the forest and the sub-alpine zone, especially affecting dwarf pine, juniper and rhododendron growth. In the Maramures Mts, all lake catchments are treeless and heavily affected by gully erosion and shallow landslides (Balteanu et al. 2016).

The basic characteristics of the sampling sites are summarized in Table 1.

### ﻿Sampling and identification

The chironomid survey was conducted during three sampling campaigns in August 2022 and July 2024 (lakes in the Maramures Mts) and in July 2023 (lakes in the Rodna Mts). Floating chironomid pupal exuviae, pupae and drowned adults were collected along the entire lake shores by skimming the water surface with a hand net (mesh size 250 μm, frame diameter 25 cm, telescopic handle).

Onshore, each sample was transferred to a labelled 100 ml plastic bottle and preserved in 4% formalin. In the laboratory, samples were placed in Petri dishes and all specimens were sorted under a stereomicroscope at a magnification of 7.5–50×.

Pupal exuviae were examined and classified to at least the genus level. All pupal exuviae of the least abundant morphotypes, as well as pharate adults and males associated with pupal skins, were mounted on microscopic slides, while at least 10 exuviae were prepared for the most abundant ones.

Berlese solution was used as the mounting medium. Chironomid pupal exuviae were identified using Langton and Visser (2003), while adults were identified with Langton and Pinder (2007a, 2007b). In some cases, more detailed keys were used, including Fittkau (1962); Reiss (1969); Säwedal (1976); and Langton et al. (2013). Species nomenclature and distribution follow Ashe and O’Connor (2009, 2012); and de Jong (2016).

All the slides and samples are archived in the Department of Biology and Environmental Studies, Faculty of Natural Sciences, Matej Bel University in Banská Bystrica, Slovakia.

### ﻿Statistical analysis

To improve the separation of *Procladius* pupal exuviae, we focused on thoracic horn parameters of the exuviae which were associated with males of *P.sagittalis* from Lakes Vartopul 1 and Vartopul 2 and compared them with those of *P.choreus* from Western Carpathian reservoirs. Our previous research showed that only thoracic horn characteristics were statistically significant for the identification of *Procladius* exuviae (Langton et al. 2013). We measured thoracic horn length, maximum breadth, and plastron plate diameter on a total of 30 thoracic horns from 15 *P.sagittalis* specimens and 81 thoracic horns from 41 *P.choreus* specimens.

For automatised classification, a decision tree classifier of Custode and Iacca (2023) was selected for its decision-making quality and interpretability.

## ﻿Results and discussion

A total of 1118 chironomid pupal exuviae, six pupae, seven pharate adults (six males, one female), 40 males and one female were collected and identified, representing 50 chironomid species/ taxa from 26 genera across 5 subfamilies. Nine Chironomidae species were recorded for the first time in Romania.

A list of all species/ taxa recorded is provided below; sampling site codes refer to Table 1; “**Pe**” after the genus name refers to a morphotype not associated with an adult by Langton (1991); * denotes the first record of a species from Romania. For detailed data on collected specimen abundance and life stages, see Suppl. materal 1.

**CHIRONOMIDAE**

**Tanypodinae**

Procladius (Holotanypus) choreus (Meigen, 1804): M1, M2, R3

*Procladius (Holotanypus) sagittalis (Kieffer, 1909): M3, M4

*Procladius (Holotanypus) simplicistilus Freeman, 1948: R5

Procladius (Holotanypus) Pe3 Langton 1991: R7, R12

*Macropelopianebulosa* (Meigen, 1804): R1, R4

*Monopelopiatenuicalcar* (Kieffer, 1918): R12

*Zavrelimyiapunctatissima (Goetghebuer, 1934): R4

**Diamesinae**

*Diamesa* Pe 5? Langton 1991: R4

Pseudodiamesa (Pseudodiamesa) nivosa (Goetghebuer, 1928): R8

**Prodiamesinae**

*Prodiamesaolivacea* (Meigen, 1818): R1, R8

**Orthocladiinae**

*Brilliabifida* (Kieffer, 1909): R9

*Bryophaenocladius* sp./ *Gymnometriocnemus* sp.: R2

*Corynoneuraceleripes* Winnertz, 1852: R3

*Corynoneuraceltica* Edwards, 1924: R4

*Corynoneuralobata* Edwards, 1924: R12

Cricotopus (Cricotopus) cf. albiforceps (Kieffer, 1916): R1

Cricotopus (Cricotopus) curtus Hirvenoja, 1973: R4

Cricotopus (Isocladius) sylvestris (Fabricius, 1794): R4

Cricotopus (Isocladius) trifasciatus (Meigen, 1810): M2

*Eukiefferiellacoerulescens* Kieffer, 1926: R4

Eukiefferiellacf.dittmari Lehman, 1972: R4

*Heterotrissocladiusmarcidus* (Walker, 1856): R2, R8, R9, R10, R11

*Krenosmittiacamptophleps* (Edwards, 1929): R4

Limnophyescf.asquamatus Andersen, 1937: M1, M2, R5, R6, R7, R8

Limnophyescf.gelasinus Saether 1990: R2

Orthocladius (Mesorthocladius) frigidus (Zetterstedt, 1838): R8

Psectrocladius (Allopsectrocladius) obvius (Walker, 1856): M4, R4

*Psectrocladius (Allopsectrocladius) platypus (Edwards, 1929): M4, R12

*Psectrocladius (Psectrocladius) oligosetus Wuelker, 1956: R3, R7, R12

Rheocricotopus (Rheocricotopus) effusus (Walker, 1856): R4

*Thienemanniella* Pe1 Langton 1991: R4

**Chironominae**

Chironomus (Chironomus) cf. aberratus Keyl, 1961: M2, M4, R3, R4, R5

Chironomus (Chironomus) cf. holomelas Keyl, 1961: R6

Chironomus (Chironomus) cf. longistylus Goetghebuer, 1921: M2, M3, M4, R3, R7

Chironomus (Lobochironomus) dorsalis Meigen, 1818: R5, R6

Chironomus (Lobochironomus) Pe2 Langton 1991: R3, R5, R6, R7

Chironomus (Chironomus) sp.: R3

*Cladopelmagoetghebueri* Spies et Saether, 2004: R12

Polypedilum (Pentapedilum) cf. uncinatum (Goetghebuer, 1921): R3

*Synendotendipeslepidus (Meigen, 1830): R5

*Synendotendipes* sp.: M3, M4, R3, R5, R6, R7, R12

*Micropsectrabodanica Reiss, 1969: M2

*Micropsectrajunci* (Meigen, 1818): R9

*Micropsectralindrothi* Goetghebuer, 1931: M2

*Micropsectranotescens (Walker, 1856): R10, R11

*Paratanytarsusaustriacus* (Kieffer, 1924): M2, R4

*Tanytarsusbathophilus* Kieffer 1911: R4, R8

*Tanytarsusgregarius* Kieffer, 1909: R1, R2, R4

*Tanytarsusmiriforceps (Kieffer, 1921): R2, R4

*Tanytarsus* Pe 4c Langton 1991/ *debilis* (Meigen, 1830): M2, M3

### ﻿Comments on new records of Chironomidae from Romania

#### ﻿ Zavrelimyia punctatissima

Taxon classificationAnimaliaDipteraChironomidae

(Goetghebuer, 1934)

5A5FFE5C-463D-579C-8309-584D7B098F16

##### Material examined.

• 6 pupal exuviae, Taul Stiol (R4), 3 July 2023.

##### Distribution.

West Palaearctic. The species is known from a few European countries: Austria, France, Germany, Italy, Norway, and Slovakia (Ashe and O’Connor 2009).

##### Habitat.

It is a cold-stenothermal species adapted to live in oligotrophic waters with high oxygen concentrations (Boggero and Lencioni 2006). Boggero (2018) considers it strictly rheophilous. The species is a typical inhabitant of the littoral, inlets and outlets of alpine lakes (Rossaro et al. 2006; Hamerlík and Bitušík 2008; Steingruber et al 2013).

##### Remarks.

Pupal exuviae closely resemble those of *Zavrelimyiahirtimana* (Kieffer, 1918), but all collected specimens exhibit very small plastron plates. The plastron plate diameter to thoracic horn length (0.054–0.055) aligns with the diagnosis of Langton and Visser (2003) for *Z.punctatissima*.

#### ﻿ Procladius (Holotanypus) sagittalis

Taxon classificationAnimaliaDipteraChironomidae

(Kieffer, 1909)

7A2889B9-5B8B-5AC4-ABE1-B1CC2FDD01A8

##### Material examined.

• 11 pupal exuviae, 1 male, Lake Vartopul 1 (M3), 1 July 2024 • 25 pupal exuviae, 2 pupae, 1 pharate adult – male, Lake Vartopul 2 (M4), 1 July 2024.

##### Distribution.

Palaearctic and Oriental. Distributed from Europe and North Africa through Iran to Japan and the Russian Far East. One record is known from China (Ashe and O’Connor 2009; de Jong 2016).

##### Habitat.

Generally, larvae of the subgenus Holotanypus are dwellers of stagnant and slow flowing waters regardless of size or volume. Langton (1991) noted that *P.sagittalis* typically occurs in shallow water under 2 m deep, which aligns with the findings from small-volume habitats (e.g., Velasco et al. 1993; Hirabayashi et al. 2004). However, the species has also been recorded from artificial ponds and reservoirs, as well as from backwaters, and large rivers (Bitušík 1993; Evrard 1994; Móra et al. 2010; Quintana et al. 2018). It should be noted that ecological information on the species could be more accurate if the identification of the preimaginal stages were more reliably resolved.

##### Remarks.

Identification of the pupal exuviae, and even adult males of Procladius (Holotanypus) is extremely challenging (Vallenduuk and Moller Pillot 2007). The extended key for exuvia (Langton et al. 2013) is not reliably applicable to *Procladius* material collected from the Maramures lakes due to the variability of the tergite armament. Notably, the distinctive “fish scale” armament typical of *P.choreus* can also appear in some specimens of *Procladius* Pe3. The parameters of the thoracic horns appear to be more reliable characteristics for identification.

Thus, we propose a model that classifies input data with 97% accuracy, achieving 100% for *P.sagittalis* and 96% for *P.choreus*. Based on the decision tree trained on our dataset, we constructed an identification key for distinguishing the aforementioned *Procladius* species (Table 2). We are aware of the tentative nature of the key and acknowledge that a larger dataset would improve the tuning and evaluation of the proposed system. Therefore, the proposed key should be used with great caution.

**Table 2.** A tentative identification key for *Procladiuschoreus* and *P.sagittalis* based on thoracic horn characteristics, using the decision tree classifier of Custode and Iacca (2023).

Question number	Question text	Result	
		Yes	No
1	Is the length of the thoracic horn ≤ 469 μm?	*P.sagittalis*	2
2	Is the diameter of the plastron plate ≤ 93 μm?	3	4
3	Is the length of the thoracic horn ≤ 478 μm?	5	*P.choreus*
4	Is the breadth of the thoracic horn ≤ 144 μm?	*P.choreus*	*P.sagittalis*
5	Is the length of the thoracic horn ≤ 476 μm?	*P.choreus*	*P.sagittalis*

#### ﻿ Procladius (Holotanypus) simplicistilus

Taxon classificationAnimaliaDipteraChironomidae

Freeman, 1948

30538B47-8AF7-55DD-83F4-AB0EDDFEB427

##### Material examined.

• 1 pharate adult – male, Lake Gargalau 3 (R5), 6 July 2023.

##### Distribution.

Palaearctic. The species was recorded only from a few countries in West and North Europe, but also from the Far East of Russia (Ashe and O’Connor 2009).

##### Habitat.

The ecological requirements of this species are not sufficiently known because of the problematic identification of the pre-imaginal stages. Generally, larvae inhabit stagnant waters, they are resistant to low pH values (Murray and Baars 2006; Perova 2008; Baars et al. 2014) and salinity (Kawai et al. 2000).

##### Remarks.

An adult male with associated exuviae confirms the presence of the species in Romania.

#### ﻿ Psectrocladius (Allopsectrocladius) platypus

Taxon classificationAnimaliaDipteraChironomidae

(Edwards, 1929)

15DC5A89-B836-545E-80EC-66F868F7B2FB

##### Material examined.

• 64 pupal exuviae, 1 pharate adult – male, Lake Vartopul 2 (M4), 1 July 2024 • 1 pupal exuviae, Taul Hardau (R3), 6 July 2023.

##### Distribution.

Palaearctic. Known from several European countries, as well as Turkey and Algeria (Ashe and O’Connor 2009).

##### Habitat.

The species is typical of small, acidic, stagnant waters in moorlands and peat bogs. In addition to tolerating low pH, it can withstand low oxygen levels in polyhumic waters; however, larvae are also found in lake littorals and small streams with slow currents (Moller Pillot 2013 and references therein). In the Western Carpathians, pupal exuviae were collected from a small, non-acid sub-alpine lake (Bitušík et al. 2006). The species is frequently recorded in temporary pools and ponds (e.g., Bazzanti et al. 1997; Puntí et al. 2007), as well as ephemeral waters (Moller Pillot 2003), indicating relatively high dispersal potential of females.

##### Remarks.

The findings indicate the humic conditions of Taul Hardau and suggest at least partial drying of Lake Vartopul 2.

#### ﻿ Psectrocladius (Psectrocladius) oligosetus

Taxon classificationAnimaliaDipteraChironomidae

Wuelker, 1956

A52EE1AC-C975-5280-9868-FAC173A110B9

##### Material examined.

• 26 pupal exuviae, Taul Hardau (R3), 6 July 2023; 59 pupal exuviae, Taul Muced (R12), 7 July 2023 • 1 pupal exuviae, Lake Gargalau 1 (R7), 6 July 2023.

##### Distribution.

Palaearctic. Recorded from several European countries ranging from the south (Sicily) to the north (Scandinavia) and from the west (Ireland) to the eastern part of Russia (Ashe and O’Connor 2009).

##### Habitat.

Cold-stenothermic species occurring in lakes in mountain regions (e.g., Laville and Vinçon 1986, Bitušík et al. 2007, Boggero 2018), although Rieradevall et al. (2007) found it in intermittent mountain headstreams. The species shows an apparent affinity for low pH humic waters (e.g., Ruse 2002; Bitušík and Svitok 2006; Moller Pillot 2013; Bitušík et al. 2020).

##### Remarks.

This finding, along with an earlier record from Ukraine (Bitušík et al. 2020), partially fills the distribution gap of the species extending from the Baltic republics across Poland to the Balkans.

#### ﻿ Synendotendipes lepidus

Taxon classificationAnimaliaDipteraChironomidae

(Meigen, 1830)

3659ACC2-31E0-5EBD-8AAB-9C0811B8D253

##### Material examined.

• 4 males, 1 female, Lake Gargalau 3 (R5), 6 July 2023.

##### Distribution.

Palaearctic. Widespread in Europe (Ashe and Cranston 1984, Moller Pillot 2009), and it has been reported from Turkey (Ozbek et al. 2018) and the Russian Far East (Orel 2016).

##### Habitat.

The species has been recorded mainly from stagnant waters regardless of size and trophic status. Lundström et al. (2010) collected adults from temporary wetlands, and there are data from lowland brooks (Ozbek et al. 2018). Like other species of the genus, it tolerates acid conditions of peatland pools (Plóciennik et al. 2018). According to Moller Pillot (2009), the larvae are miners in the tissues of *Nupharlutea*. However, they evidently utilize other types of littoral vegetation, such as sedges, since *N.lutea* does not occur in the studied lakes.

##### Remarks.

Species of the genus *Synendotendipes* are indistinguishable as pupal exuviae, so it is not possible to confirm if *Synendotendipes* pupal exuviae recorded in other lakes also belong to *S.lepidus.*

#### ﻿ Micropsectra bodanica

Taxon classificationAnimaliaDipteraChironomidae

Reiss, 1969

799F3DDE-D436-50AD-9970-2B31F5F4C577

##### Material examined.

• 1 male, Lake Vinderel (M2), 1 July 2023.

##### Distribution.

Palaearctic. The species has so far been recorded from only a few countries, such as Germany, Austria, and Portugal, with its occurrence in Corsica and Slovakia not yet confirmed (Moubayed-Breil and Ashe 2012; Novikmec et al. 2015).

##### Habitat.

Ecological requirements of the species are still inadequately understood. Reiss (1969) considered the species (together with *M.attenuata*) as cold stenothermic and polyoxybiontic, typically inhabiting mosses on stones in springs and the upper stretches of streams (see also Langton and Visser 2003). Records of the pupal exuviae of *M.attenuata*/*bodanica* in the Western Carpathians come from headwater streams (one even artificially modified) with stony bottoms but without moss growths (Novikmec et al. 2015). It can be assumed that the collected adult male comes from a spring or small stream flowing in Lake Vinderel.

##### Remarks.

Since the pupal exuviae of *M.bodanica* are indistinguishable from those of *M.attenuata* (Langton and Visser 2003), the first record of *M.bodanica* based on an adult male in Romania is particularly valuable.

#### ﻿ Micropsectra notescens

Taxon classificationAnimaliaDipteraChironomidae

(Walker, 1856)

58C99E7F-6BE9-54C6-9EC8-758B77861E14

##### Material examined.

• 26 pupal exuviae, Lake Buhaescu 2 (R10), 5 July 2023 • 22 pupal exuviae, Lake Buhaescu 3 (R11), 5 July 2023.

##### Distribution.

Palaearctic. Widespread in Europe including the Canary Islands (Langton and Visser 2003); also recorded in Morocco (Kettani and Langton 2011).

##### Habitat.

Traditionally, the species is considered cold stenothermic and polyoxybiontic (Säwedal 1976). It has been documented in mountain, boreal and woodland springs and spring brooks (Orendt 2000; Ilmonen et al. 2009; Lencioni et al. 2012), as well as alpine lakes and ponds (Bitušík et al. 2006; Oertli et al. 2010; Lods-Crozet et al. 2012). However, data from low-altitude streams suggest a wider temperature tolerance (Móra and Szivák 2012). Its presence in temporary habitats, such as fountains (Oboňa et al. 2017) and temporarily flooded wetlands (Lundström et al. 2010), indicates a high distribution potential of females.

##### Remarks.

The presence of *M.notescens* in Romania has already been reported by Säwedal (1976) based on two males collected by Andrzej Kownacki from the Fagaras Mts. However, the species is not listed in the latest checklist of Romanian Chironomidae (Tatole 2023).

#### ﻿ Tanytarsus miriforceps

Taxon classificationAnimaliaDipteraChironomidae

(Kieffer, 1921)

79090BE0-B43A-5757-ACFD-FCCDC9265525

##### Material examined.

• 13 pupal exuviae, 1 adult – male, Taul Stiol (R4), 3 July 2023 • 110 pupal exuviae, 1 pharate adult – male, 1 adult – male, Lake Lala Mica (R2), 4 July 2023.

##### Distribution.

Holarctic. The species is widespread across Europe, primarily in northern and western countries (Ashe and Cranston 1984; de Jong 2016), with recent records in Poland, Montenegro and European Russia (Gilka and Dominiak 2007; Krasheninnikov 2014; Gadawski et al. 2022). It is also known from Canada (de Jong 2016); and the Far East (Orel 2018).

##### Habitat.

Current data indicate that this species is a limnobiont inhabiting lakes mainly at high altitudes and high latitudes (except for Lake Skadar), suggesting a preference for low temperatures (e.g., Verneaux and Aleya 1999).

##### Remarks.

The species exhibits symptoms of glacial relictualism as already suggested by Reiss and Fittkau (1971) and Reiss (1984).

The collection of floating chironomid pupal exuviae from the lakes in this study provides an excellent basis for the chironomid inventory of the area. For species identification, exuviae are sometimes even more useful than adults (Prat et al. 2016). However, it should be noted that our species inventory from a “snapshot” survey cannot be comprehensive, as not all species present in a site emerge simultaneously. Even though the collection was conducted during a period suitable for recording most species (Wilson and Ruse 2005; own data), we believe that the absence of cold-stenothermic species/ genera in our collection is due to their early spring emergence.

Compared to some Central and East European countries, such as Hungary, Ukraine, Czechia, Slovakia, and Poland, the Romanian chironomid fauna is relatively well-studied. The latest checklist of the family from Romania (Tatole 2023) includes 517 species, with recent records of nine additional species raising this total to 526. This number could be even higher if species within the genus *Limnophyes* and some *Chironomus* species could be reliably identified.

A detailed examination of the chorological data in the aforementioned checklist reveals a lack of records from the Rodna and Maramures Mountains. Chironomids are also absent from the list of Diptera collected in Maramures Mountains Nature Park (Parvu 2008). The only available information on chironomids associated with the studied lakes comes from the sediment core of Lake Taul Muced, where subfossil larval remains were identified to morphotype level (Diaconu et al. 2017).

Here, we provide the first information about chironomid occurrence within the protected areas of Maramures Mountains Nature Park and Rodna Mountains National Park, offering potential value to use by the administrations of both parks.

## Supplementary Material

XML Treatment for Zavrelimyia punctatissima

XML Treatment for Procladius (Holotanypus) sagittalis

XML Treatment for Procladius (Holotanypus) simplicistilus

XML Treatment for Psectrocladius (Allopsectrocladius) platypus

XML Treatment for Psectrocladius (Psectrocladius) oligosetus

XML Treatment for Synendotendipes lepidus

XML Treatment for Micropsectra bodanica

XML Treatment for Micropsectra notescens

XML Treatment for Tanytarsus miriforceps

## ﻿Additional information

### Author ORCIDs

Peter Bitušík https://orcid.org/0000-0002-8439-4582

Veronika Slobodníková https://orcid.org/0000-0003-4098-5791

Milan Novikmec https://orcid.org/0000-0002-5192-4575

Adam Dudáš https://orcid.org/0000-0001-5517-9464

Ladislav Hamerlík https://orcid.org/0000-0002-0803-8981

### Data availability

All of the data that support the findings of this study are available in the main text or Supplementary Information.

## Supplementary materials

Supplementary material 1 List of chironomid taxa collected from studied lakes in the Maramures Mts. and the Rodna Mts. Data type docx Explanation note Sampling site codes refer to codes in Table 1; numbers refer to number of chironomid pupal exuviae collected; P – pupa, M – male, F – female (in case of adult specimens), PhM – pharate adult male, PhF – pharate adult female, * – first record of species for Romania. https://binary.pensoft.net/file/1293698This dataset is made available under the Open Database License (http://opendatacommons.org/licenses/odbl/1.0/). The Open Database License (ODbL) is a license agreement intended to allow users to freely share, modify, and use this Dataset while maintaining this same freedom for others, provided that the original source and author(s) are credited.Peter Bitušík, Veronika Slobodníková, Milan Novikmec, Adam Dudáš, Ladislav Hamerlík
