# Supplementary material for: ﻿Chironomidae (Diptera) from mountain lakes of the Eastern Carpathians, Romania: First records and insight into diversity
Source: Zookeys. 2025 Mar 28;1233:107–23. doi: 10.3897/zookeys.1233.142856 (PMC11971640; doi:10.3897/zookeys.1233.142856)
Supplement: Supplementary material 1 — List of chironomid taxa collected from studied lakes in the Maramures Mts. and the Rodna Mts. [file zookeys-1233-107_article-142856__-s001.docx]

Supplementary material. List of chironomid taxa collected from studied lakes in the Maramures Mts. and the Rodna Mts. Sampling site codes refer to codes in Tab. 1; numbers refer to number of chironomid pupal exuviae collected; **P** – pupa, **M** – male, **F** – female (in case of adult specimens), **PhM** – pharate adult male, **PhF** – pharate adult female, * – first record of species for Romania.

|  | **M1** | **M2** | **M3** | **M4** | **R1** | **R2** | **R3** | **R4** | **R5** | **R6** | **R7** | **R8** | **R9** | **R10** | **R11** | **R12** |
| --- | --- | --- | --- | --- | --- | --- | --- | --- | --- | --- | --- | --- | --- | --- | --- | --- |
| **Tanypodinae** |  |  |  |  |  |  |  |  |  |  |  |  |  |  |  |  |
| *Procladius* (*Holotanypus*) *choreus* (Meigen, 1804) | 1 | 3 |  |  |  |  | 9 |  |  |  |  |  |  |  |  |  |
| **Procladius* (*Holotanypus*) *sagittalis* (Kieffer, 1909) |  |  | 11, 1M | 25, 2P, 1PhM |  |  |  |  |  |  |  |  |  |  |  |  |
| **Procladius* (*Holotanypus*) *simplicistilus* Freeman, 1948 |  |  |  |  |  |  |  |  | 1PhM |  |  |  |  |  |  |  |
| *Procladius* (*Holotanypus*) Pe3 Langton 1991 |  |  |  |  |  |  |  |  |  |  | 1 |  |  |  |  | 19 |
| *Macropelopia* *nebulosa* (Meigen, 1804) |  |  |  |  | 4 |  |  | 10 |  |  |  |  |  |  |  |  |
| *Monopelopia* *tenuicalcar* (Kieffer, 1918) |  |  |  |  |  |  |  |  |  |  |  |  |  |  |  | 5, 4P |
| **Zavrelimyia* *punctatissima* (Goetghebuer, 1934) |  |  |  |  |  |  |  | 6 |  |  |  |  |  |  |  |  |
| **Diamesinae** |  |  |  |  |  |  |  |  |  |  |  |  |  |  |  |  |
| *Diamesa* Pe 5? Langton 1991 |  |  |  |  |  |  |  | 2 |  |  |  |  |  |  |  |  |
| *Pseudodiamesa* (*Pseudodiamesa*) *nivosa* (Goetghebuer, 1928) |  |  |  |  |  |  |  |  |  |  |  | 2 |  |  |  |  |
| **Prodiamesinae** |  |  |  |  |  |  |  |  |  |  |  |  |  |  |  |  |
| *Prodiamesa* *olivacea* (Meigen, 1818) |  |  |  |  | 2 |  |  |  |  |  |  | 5 |  |  |  |  |
| **Orthocladiinae** |  |  |  |  |  |  |  |  |  |  |  |  |  |  |  |  |
| *Brillia* *bifida*(Kieffer, 1909) |  |  |  |  |  |  |  |  |  |  |  |  | 1 |  |  |  |
| *Bryophaenocladius* sp./ *Gymnometriocnemus* sp. |  |  |  |  |  | 1 |  |  |  |  |  |  |  |  |  |  |
| *Corynoneura* *celeripes* Winnertz, 1852 |  |  |  |  |  |  | 1M |  |  |  |  |  |  |  |  |  |
| *Corynoneura celtica* Edwards, 1924 |  |  |  |  |  |  |  | 1 |  |  |  |  |  |  |  |  |
| *Corynoneura lobata* Edwards, 1924 |  |  |  |  |  |  |  |  |  |  |  |  |  |  |  | 28, 1M |
| *Cricotopus* (*Cricotopus*) cf. *albiforceps* (Kieffer, 1916) |  |  |  |  | 2 |  |  |  |  |  |  |  |  |  |  |  |
| *Cricotopus* (*Cricotopus*) *curtus* Hirvenoja, 1973 |  |  |  |  |  |  |  | 1 |  |  |  |  |  |  |  |  |
| *Cricotopus* (*Isocladius*) *sylvestris* (Fabricius, 1794) |  |  |  |  |  |  |  | 17 |  |  |  |  |  |  |  |  |
| *Cricotopus* (*Isocladius*) *trifasciatus* (Meigen, 1810) |  | 2 |  |  |  |  |  |  |  |  |  |  |  |  |  |  |
| *Eukiefferiella* *coerulescens* Kieffer, 1926 |  |  |  |  |  |  |  | 1 |  |  |  |  |  |  |  |  |
| *Eukiefferiella* cf. *dittmari* Lehman, 1972 |  |  |  |  |  |  |  | 2 |  |  |  |  |  |  |  |  |
| *Heterotrissocladius* *marcidus* (Walker, 1856) |  |  |  |  |  | 2 |  |  |  |  |  | 11 | 4 | 4, 3M | 2, 1M |  |
| *Krenosmittia* *camptophleps* (Edwards, 1929) |  |  |  |  |  |  |  | 8 |  |  |  |  |  |  |  |  |
| *Limnophyes* cf. *asquamatus* Andersen, 1937 | 4, 1M | 1 |  |  |  |  |  |  | 1M? | 3 | 3, 1M | 1 |  |  |  |  |
| *Limnophyes* cf. *gelasinus* Saether 1990 |  |  |  |  |  | 1 |  |  |  |  |  |  |  |  |  |  |
| *Orthocladius* (*Mesorthocladius*) *frigidus* (Zetterstedt, 1838) |  |  |  |  |  |  |  |  |  |  |  | 1 |  |  |  |  |
| *Psectrocladius* (*Allopsectrocladius*) *obvius* (Walker, 1856) |  |  |  | 14 |  |  |  | 1 |  |  |  |  |  |  |  |  |
| **Psectrocladius* (*Allopsectrocladius*) *platypus* (Edwards, 1929) |  |  |  | 64, 1 PhM |  |  |  |  |  |  |  |  |  |  |  | 1 |
| **Psectrocladius* (*Psectrocladius*) *oligosetus* Wuelker, 1956 |  |  |  |  |  |  | 26 |  |  |  | 1 |  |  |  |  | 59 |
| *Rheocricotopus* (*Rheocricotopus*) *effusus* (Walker, 1856) |  |  |  |  |  |  |  | 1 |  |  |  |  |  |  |  |  |
| *Thienemanniella* Pe1 Langton 1991 |  |  |  |  |  |  |  | 2 |  |  |  |  |  |  |  |  |
| **Chironominae** |  |  |  |  |  |  |  |  |  |  |  |  |  |  |  |  |
| *Chironomus* (*Chironomus*) cf. aberratus Keyl, 1961 |  | 2 |  | 3 |  |  | 1 | 1 | 20 |  |  |  |  |  |  |  |
| *Chironomus* (*Chironomus*) cf. *holomelas* Keyl, 1961 |  |  |  |  |  |  |  |  |  | 1 |  |  |  |  |  |  |
| *Chironomus* (*Chironomus*) cf. *longistylus* Goetghebuer, 1921 |  | 3 | 5 | 34 |  |  | 6 |  |  |  | 1 |  |  |  |  |  |
| *Chironomus* (*Lobochironomus*) Pe2 Langton 1991 |  |  |  |  |  |  | 16 |  | 5 | 33 | 4 |  |  |  |  |  |
| *Chironomus* (*Lobochironomus*) *dorsalis* Meigen, 1818 |  |  |  |  |  |  |  |  | 1M | 17 M |  |  |  |  |  |  |
| *Chironomus* (*Chironomus*) sp. |  |  |  |  |  |  | 1 |  |  |  |  |  |  |  |  |  |
| *Cladopelma* *goetghebueri* (Spies et Saether, 2004)) |  |  |  |  |  |  |  |  |  |  |  |  |  |  |  | **7** |
| *Polypedilum* (*Pentapedilum*) cf. *uncinatum* (Goetghebuer, 1921) |  |  |  |  |  |  | 1 |  |  |  |  |  |  |  |  |  |
| **Synendotendipes* *lepidus* (Meigen, 1830) |  |  |  |  |  |  |  |  | 4M, 1F |  |  |  |  |  |  |  |
| *Synendotendipes* sp. |  |  | 14 | 1 |  |  | 9 |  | 41 | 28 | 1 |  |  |  |  | 21 |
| **Micropsectra* *bodanica* Reiss, 1969 |  | 1M |  |  |  |  |  |  |  |  |  |  |  |  |  |  |
| *Micropsectra* *junci* (Meigen, 1818) |  |  |  |  |  |  |  |  |  |  |  |  | 14, 2M |  |  |  |
| *Micropsectra* *lindrothi* Goetghebuer, 1931 |  | 14, 1PhF |  |  |  |  |  |  |  |  |  |  |  |  |  |  |
| **Micropsectra* *notescens* (Walker, 1856) |  |  |  |  |  |  |  |  |  |  |  |  |  | 26 | 22, 1M? |  |
| *Paratanytarsus* *austriacus* (Kieffer, 1924) |  | 1 |  |  |  |  |  | 54 |  |  |  |  |  |  |  |  |
| *Tanytarsus* *bathophilus* Kieffer 1911 |  |  |  |  |  |  |  | 74 |  |  |  | 60, 2M |  |  |  |  |
| *Tanytarsus* *gregarius* Kieffer, 1909 |  |  |  |  | 84, 2PhM | 1 |  | 1M |  |  |  |  |  |  |  |  |
| **Tanytarsus* *miriforceps* (Kieffer, 1921) |  |  |  |  |  | 110, 1PhM, 1M |  | 13, 1M |  |  |  |  |  |  |  |  |
| *Tanytarsus* Pe 4c Langton 1991/ *debilis* (Meigen, 1830) |  | 4 | 7 |  |  |  |  |  |  |  |  |  |  |  |  |  |
